# The infiltration of microplastics in human systems: Gastrointestinal accumulation and pathogenic impacts

**DOI:** 10.1016/j.heliyon.2025.e42606

**Published:** 2025-02-12

**Authors:** Pramesh Sinha, Vaishali Saini, Nidhi Varshney, Rajan Kumar Pandey, Hem Chandra Jha

**Affiliations:** a Infection Bioengineering Group, Department of Biosciences and Biomedical Engineering, Indian Institute of Technology Indore, Simrol, Indore, 453552, Madhya Pradesh, India; b Department of Medical Biochemistry and Biophysics, Karolinska Institute, 17165, Stockholm, Sweden; c Centre for Rural Development and Technology, Indian Institute of Technology Indore, Indore, Madhya Pradesh, India

**Keywords:** Microplastics, Barrier disruption, Toxicology, Gut dysbiosis, *Helicobacter pylori*

## Abstract

Microplastic particles have become ubiquitous in various ecosystems due to a drastic increase in plastic use and its consequent litter. The biological effects of these plastic particles on aquatic fauna are well-documented. However, the study of their accumulation and subsequent impact on terrestrial flora and fauna is in its initial stages. Furthermore, the favorable surface provided by plastics lodges various harmful substances and pathogens known to cause varied effects on human physiology. Notably, the entry of plastics into the gastrointestinal tract can result in various ailments, including dysbiosis of gut microflora and microbial biodiversity. Moreover, similar physiological ailments have been observed in humans due to the action of pathogenic microorganisms. Therefore, in this review, we aim to explore the relationship and possible amplification of pathogenesis due to the ability of plastic particles to provide favorable surfaces for the absorption and biofilm formation of such microorganisms. Additionally, there exists a possibility of carcinogenesis due to the coexistence of pathogenic microbes and micro-and nanoplastics due to their synergistic effects leading to severe human ailments.

## Highlights

• Microplastic pollution surmounts harmful effects on human and aquatic biosystems.
• Intestinal barrier disruption is common route for both microplastic and pathogen entry.
• Microplastics form biofilm that provides a potential surface for pathogens to grow.
• Pathogen-microplastic biofilm might reach extra-gastric regions to enhance disease spread.

## Graphical abstract

**Figure:**
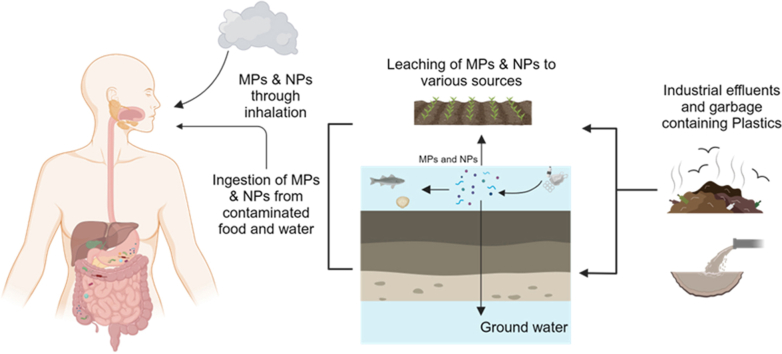

## 1 Introduction

Plastic products are widely used for their various properties, such as strength, resistance to degradation and heat, durability, and cost-effectiveness. Consequently, the dependence on plastics has steadily increased in industry, agriculture, and daily use [1]. The global rise in plastics production has resulted in increased garbage disposal and an alarming increase in ecological pollution. Significant sources of plastic pollution include particulate dust from industrial production and effluents from both industrial and domestic origin. Many plastic polymers contribute to environmental pollution, including polystyrene (PS), polyethylene (PE), high-density polyethylene (HDPE), polylactic acid (PLA), polyurethane (PU), polyethylene terephthalate (PET), polyamides (PA), polypropylene (PP), polyvinyl chloride (PVC), polyester (PES) and styrene acrylate (SA). The small-sized particles in dust from production facilities give rise to primary plastics. Additionally, the plastic pollutants in effluent from various sources get exposed to natural forces such as UV radiation, chemicals, and abrasion, which fragment plastics into micro- and nano-sized plastics, also known as secondary plastics [2]. Plastics ≤5 mm in diameter are considered microplastics (MPs), whereas plastics ranging from 1 nm to 1 μm are nanoplastics (NPs) [3,4]. MPs exist in various types, such as fragments, fibers, beads, granules, pellets, flakes, and spheroids [5]; the most common varieties present in environmental waste are fibers and fragments [6,7]. The accumulation and effects of plastics’ entry into aquatic ecosystems and their impact on marine organisms, including gastric injury, gut dysbiosis, and inflammation, are well documented [8,9]. However, the study of the accumulation and effects of plastics on humans is still in the initial stages.

The primary pathways for entry of MPs and NPs in humans are inhalation, direct or indirect ingestion, and dermal contact [10]. The consequential entry of MPs in humans through the consumption of aquatic animals is the major contributor to plastic uptake. Other sources of MP ingestion include consuming contaminated crops and beverages [11]. Moreover, the entry of plastics through cosmetics and pesticides containing MPs is also a reason for concern [12]. The specific concentrations of different polymers vary with changes in the location; the most abundant polymers in the Indian subcontinent are PE, HDPE, PET, and PS.

Plastics’ persistent presence and consequent entry into humans results in its accumulation. Initially, the MP accumulation site in humans was hypothesized to be the liver, followed by the kidney, and ultimately, the gut [13]; however, recent studies have shown that the primary site of accumulation was the gastrointestinal tract and lymph nodes, particularly the small intestine, followed by the liver and spleen [[13], [14], [15], [16]].

Additionally, the large, inert, and hydrophobic surface of MPs acts as a “raft” for hydrophobic pollutants, toxins, and microbes, which can be attributed to its adsorption properties [17], consequently facilitating the invasion of multiple opportunistic pathogens. Moreover, certain carcinogenic chemicals have been observed to be adsorbed on the MP surface as polycyclic aromatic hydrocarbons (PAH), consequently increasing their bioavailability and ingestion [18]. The known effects of pollutants and pathogens' entry into the human gastrointestinal tract are disruption of the mucous barrier, intestinal barrier, and inflammation [19]. Moreover, the persistence of these effects leads to several disorders, such as gut dysbiosis, also known as dysbacteriosis, a condition in which there is disruption or reorganization of the gut microbiota, resulting in a severe imbalance between beneficial and harmful microbes [20] and leaky gut syndrome, which refers to the permeability of the intestine due to disruption of gut microbiota. In this review, we have discussed the relationship of MPs with pathogenic microorganisms and carcinogenesis.

## 2 Plastic additives: accumulation and toxicity in different organisms

Plastics are synthetic polymeric compounds obtained from petrochemical sources; other chemicals can also be added to enhance their performance. The usual method of producing plastics is by heating the polymer granules to a molten state and adding additives that give a particular property to the plastic polymer; for example, foamed PS can be produced by adding a blowing agent during the polymerization process. Introducing these chemicals significantly alters the bulk properties of a plastic polymer; however, additives are not chemically bonded to the polymer. The most common additive chemical compounds used in the polymerization of plastics are fillers, anti-aging agents, colorants, plasticizers, softeners, lubricants, flow promoters, impact modifiers, flame retardants, and blowing agents [21]. Due to the inclusion of additives, properties such as stability under processing conditions, non-toxicity, and cost-effectiveness can be achieved [22]. The possibility of adverse effects because of additives is due to the leaching or bleeding of compounds mentioned above in the environment and the ingestion of plastics by organisms such as fishes, sea birds, etc. Additionally, these additives accumulate in the body of organisms and travel to higher trophic levels through the food chain [23,24]. The known effects of plastic additives are carcinogenesis in different organisms, decrease in reproductive health and general toxicity to different organs in lower vertebrates and mammals [25]. Lastly, endocrine-disrupting effects have also been reported, which have been linked to obesity, cardiovascular disorders, reproductive disorders and long-term ailments like cancer [26].

## 3 Entry and accumulation of microplastics in different organisms

The increase of plastics in garbage dumps and effluents from various sources poses a significant threat to multiple species of organisms in varied ecosystems. Moreover, the accumulation of plastics in the form of MPs is evident in many biological systems of varied ecosystems due to their small size and high availability [27,28]. This accumulation of plastics in the ecosystem and their entry into its inhabitants can lead to biomagnification in organisms of higher trophic levels, such as humans.

### 3.1 Plants

Plastics' innate durability and stability make them ideal for accumulation in terrestrial ecosystems. The concentration of tiny plastic particles can reach up to 6.7 % of soil weight in highly polluted areas, depending on the source of pollution and the variety of particles [29]. For example, PP and PE are the primary types of pollutants in soil affected by the application of sludge, plastic films, sewage, and irrigation of plastic-rich water [30]. Additionally, soil accumulates more plastics than aquatic ecosystems [31], consequently affecting plants, a significant part of the terrestrial ecosystem. The possible impacts of plastics on terrestrial plants pose an increasing concern due to the critical role that plants play in terrestrial ecosystems and the persistent release of plastics from various sources such as wastewater treatment plants (WWTP) [32].

Moreover, the permeation of plastics into seeds, fruits, roots, stems, leaves and plant cells has also been reported to a certain extent [33]. Apoplastic movement of water allows passage of MPs through the immature section of roots to casparian strips and bands within the radial walls, while the symplastic pathway facilitates the endocytosis of MPs; these are the primary pathways for MPs’ transport to the upper parts through vascular networks [34]. Symplastic translocation is influenced by the size exclusion limit of plasmodesmata between neighboring cells, while apoplastic translocation is governed by the size exclusion limit (5–20 nm) of the cell wall. Transpirational pull or suction force is also responsible for the uptake of plastics as aggregates [[35], [36], [37]]. Furthermore, the roots of higher plants are highly susceptible to the accumulation of MPs on their surface, hindering the exchange of nutrients and water and inducing phytotoxicity and oxidative stress [37,38].

The perpetual presence of MPs in soil hampered the diversity of soil biota and its symbiotic relationship with terrestrial flora, mainly observed in agroecosystems [39]. Depending on the type, various detrimental effects have been identified, such as delay in germination rates, hindered growth and photosynthesis, decrement in water and nutrient uptake, and decline in biomass [[40], [41], [42], [43], [44]]. Moreover, a plant-based diet has been integral to human nutrition, providing essential nutrients and promoting health. Consequently, the sustenance of plastics in plant systems and their continual uptake by humans can lead to significant bioaccumulation of MPs in humans due to a predominant plant diet.

### 3.2 Aquatic organisms

Plastics account for 60–80 % of marine trash and get broken down into MPs by environmental factors such as UV radiation, seawater, and aquatic biota; this process of breakdown is known as biodegradation, which is primarily carried out by microbes of the ecosystem [45]. The MP particles enter aquatic fauna through direct or indirect ingestion due to the indiscriminate feeding mechanism of marine biota [46]. The reports of MP ingestion are most prevalent in different species of bivalves, fishes, aquatic mammals, and seabirds [47]. MPs detected in fish species exhibit differences in shape, size, color, and type of polymer, but the most abundant are fibers and fragments, which follow their trend of presence in global waters [48,49]. After consumption, most MPs are retained in the digestive tract, especially in the stomach and intestine of the fish [50]. Moreover, these plastics can also adhere and translocate to other tissues, such as gills, muscles, and liver [51]. Reports of finer particles entering the lymphatic and circulatory systems across the living cells, especially the intestinal epithelium, through either endocytosis or paracellular persorption via dendritic cells, resulting in whole-body distribution, have also been observed [50,52].

The result of MP ingestion is primarily physical damage such as obstruction and internal abrasion entanglement of feeding appendages and obstruction and/or corrosion of internal organs [53]. These factors lead to decreased feeding, poor health, injury, and even death [54]. According to Lu et al., ingestion of MPs could also induce inflammatory responses in fish, change their level of metabolites, and disturb their innate immune system [55,56]. Additionally, predatory performance and efficiency are also affected by the presence of these pollutants in the same environment as the prey [57]. Therefore, a pattern of accumulation similar to the terrestrial ecosystem can be inferred from the evidence and plausible biomagnification in organisms of higher trophic levels.

### 3.3 Humans

Humans are exposed to MPs through food consumption, drinking water, and even through inhalation of air. Besides this, MPs are also found in cosmetic products such as facial scrubs and washes. The minuscule size of these particles makes the entry of MPs into any environment a rudimentary task. MPs are also used extensively in cosmetics due to their exuviating and abrading properties [58]. The average amount of plastic particles consumed through food annually was evaluated to be around 39,000–52,000 particles or around 0.2–6035 items.g^−1^ [59,60]. Following oral and inhalation exposure, several studies highlight the possibility of translocation and uptake of MPs and NPs into the human body [61]. Leslie et al. elucidated the presence of such plastics in blood, reaching a concentration of 1.6 μg/ml [62]. After entering the body, plastic particles can circulate via the bloodstream and enter secondary organs by overcoming the primary tissue barriers. Research conducted *in vitro* has demonstrated that carboxylated nanoparticles can penetrate as well as adsorb to red blood cells (RBCs) due to a combination of hydrogen bonding, hydrophobic, electrostatic, and van der Waals forces between the polystyrene and the RBCs [63,64]. The nanoparticles avoid swift expulsion by the liver and spleen through this hitchhiking mechanism, increasing their circulation time. These particles can reach secondary barriers such as the placental and blood-brain barriers (BBB) through the bloodstream [65,66]. The proof of MP ingestion was found through the analysis of stool samples through the use of spectroscopy techniques such as Raman spectroscopy, and the amount estimated was around 28–41 items/g [67,68].

Plastics in micro-sized fibers have been detected in lung tissue, indicating the possible translocation of MPs and NPs into the human body through particle inhalation, even in the previous century [69]. Ingestion of MPs has been suggested as a probable cause of oxidative stress, pro-inflammatory responses, and toxicity [70]. NPs are deemed to be considerably more hazardous as they can enter cells and travel into tissues and organs due to their smaller size [71]. In conclusion, the hazardous effect of plastics in the form of MPs and NPs indicates many more ailments due to their persistence and accumulation in the human body.

## 4 Effect of plastic ingestion: acute and chronic disorder

The evidence of harmful effects is thoroughly studied in aquatic organisms, as plastic litter is a significant pollutant in marine ecosystems. However, the impact of plastic intake on terrestrial critters and mammals needs to be studied in depth. This lack of evidence is due to the lower exposure of terrestrial organisms than aquatic fauna [14]. The inherent inert nature of the plastic particles can be a factor for the reduced effect of plastic ingestion in higher organisms. Nevertheless, a few consequences have been reported due to prolonged exposure to these particles. The subsequent subsections will explore acute and chronic disorders caused by plastics.

### 4.1 Acute disorder - inflammation

The preliminary studies on the exposure of MPs and NPs in biologically significant doses indicate observable changes in the gut microbiome. The consequences of these changes include inflammation in the human gut and promotion of human pathobionts such as *Enterobacteriaceae* and *Desulfovibrionaceae* in mucosal and luminal compartments at the expense of beneficial bacteria such as *Akkermansiaceae* and *Christensenellaceae* [72]. Another effect of such exposure can be gastric injury and inflammation, as reported by Tong et al. in mice models [73]. The probable pathway for inflammation can be either through cytokines such as interleukin-6 (IL-6) and interleukin-8 (IL-8), released by activated immune cells such as macrophages, neutrophils or dendritic cells or phagocytosis of these particles which might lead to reactive oxygen species (ROS) production and consequently apoptosis [74,75].

Reports of bacteria-rich biofilms on the surface of MPs have been identified in marine ecosystems [76,77]; this mitigates the idea that bacteria can adhere to the surface of MPs *in situ* and may provide a scaffold for nascent biofilm formation. A similar situation may also occur in the gastrointestinal tract, especially in the colon, facilitated by the attachment of amino acids and protein to the negatively charged surface of MPs [78]. Furthermore, because of their high surface area-to-volume ratio, which is a distinguishing feature of nanoparticles, they may have additional harmful consequences in cases where other contaminants, including persistent organic pollutants, are adsorbed and go through bioaccumulation and bioamplification processes [79].

### 4.2 Chronic disorder - carcinogenesis

Carcinogenesis can occur in either a genotoxic or non-genotoxic manner. Direct DNA damage by mutagens can result in genotoxic carcinogenesis [80]. MPs can transport surface-associated hazardous substances acquired through the environment, such as persistent organic pollutants (POPs), polycyclic aromatic hydrocarbons (PAHs), and hydrophobic organic chemicals (HOCs) due to their hydrophobic surface and significant surface-to-volume ratio [18,81,82]. These MP-mediated exposures create health hazards, including directly transporting hazardous compounds associated with MP to the underlying epithelium [83].

To identify health risks, it is essential to comprehend potential human exposure. *In vivo*, NPs can cause cytotoxicity, pro-inflammatory activity, or the generation of ROS [84]. NPs can trigger inflammatory reactions or mediate oxidative stress, as demonstrated by several *in vitro* experiments involving human cell lines [85,86]. According to Forte et al., in experiments involving human gastric adenocarcinoma cells (AGS) and unmodified polystyrene nanoparticles with a diameter of 44 nm, the genes encoding IL-6 and IL-8 got highly upregulated. These studies suggested that the induction of pro-inflammatory responses by NPs doesn't have to be charge-driven, as many studies have suggested. Instead, they could be a material-based or straightforward particle occurrence issue [85]. Two recent reviews explored the carcinogenic risk of MPs, and both indicated the possibility of an association [10,87]. Domenech and colleagues note that most research has used short-term rodent trials or *in vitro* models, making it easier to derive definitive inferences [10].

Moreover, a study by McCarthy et al. reported that human lung cells exposed to polystyrene nanoparticles activated ion channels. Applying 20 nm carboxylated polystyrene particles stimulated the release of Cl^−^ and HCO^3−^ and activated basolateral K^+^ channels, resulting in continuous short-circuit currents that lead to respiratory muscle weakness and exacerbate any underlying condition. These results highlight the possibility that polystyrene nanoparticles might influence physiological processes and the function of epithelial cells [88]. Additionally, Fuchs et al. studied the polarisation of human macrophages towards the M1 or M2 phenotype under the influence of 120 nm amino-modified and carboxylate polystyrene particles. Their findings revealed that the expression of M1 markers CD86, TNF-α, NOS2, and IL-1β were unaltered. However, for the M2 phenotypes, both the nanoparticles reduced the release of IL-10 and the expression of scavenger receptors CD200R and CD163. The carboxylated particles increased the amount of protein in M1 and M2, cytokine TGF-*β*1 released by the M1 phenotype, and the level of ATP in the M2 phenotype but did not influence the phagocytosis property of M2 types. However, the amino-modified particles hindered the phagocytosis of *Escherichia coli* by both the phenotypes M1 and M2 macrophage [89]. Furthermore, a study by Deng et al., exploring the effects of polystyrene MPs on Sprague Dawley (SD) rats indicated an association with carcinogenesis due to oxidative stress, genotoxicity, mitochondrial damage and the β-catenin/YAP signaling cascade in gastric tissues and GES-1 cells [90]. In conclusion, the invasion of MPs and their subsequent effects, such as genotoxicity due to oxidative stress, provide some preliminary clues for investigating MP-related carcinogenic effects.

## 5 Possible pathogenesis of MPs & NPs

The pathogenic potential of MPs and NPs is alarming. It can be attributed to distinct causes, including the dissipation of plastic additives, as they are not chemically attached to the polymeric molecule. Additionally, the innate nature of polymers to degrade into smaller particles and the possible adherence of pollutants such as PAHs have also been identified as the primary modulators of human ailment [91]. The sustainable nature of these plastics augments the ability of pathogenic microbes to thrive in the host as they provide a surface for growth. The spread of MPs and NPs to different organs causing varied effects, such as ROS production and inflammation, as evidenced by studies using various human cell lines (Table 1).

**Table 1:** Overview of recent advances in the effects of plastics on human cell lines.

**Size**	**Target/Cell line**	**Finding**	**Reference**
20 nm	Human lung adenocarcinoma (Calu3)	Activation of ion transport	[88]
44 nm	Human gastric adenocarcinoma cells (AGS)	Induced Upregulation of IL6 & IL8	[85]
50, 100 nm	Human colon adenocarcinoma cells (HT29-MTX), Human colon carcinoma cells (Caco-2), human intestine microfold cells	Size dependency regarding particle translocation	[86]
116 nm	Human lung carcinoma cells (A549)	Cellular uptake	[92]
120 nm	Human monocyte macrophages	Reduced expression of scavenger receptor CD163 and CD200R, and the release of IL-10	[89]
0.1, 5 μm	Human colon carcinoma cells (Caco-2)	Cytotoxicity, mitochondrial depolarisation and inhibition of ABC transporter activity	[93]
1 μm	Human embryonic kidney cells & liver (HEK 293, HEPG2)	Internalisation led to a decline in metabolism and proliferation and oxidative stress	[94]

### 5.1 Cardiovascular system

Recently, cardiovascular disease (CVD) has been identified as one of the significant causes of ailment in humans. Many studies have demonstrated that many small particles have toxicity to the myocardial tissue, causing cardiovascular dysfunction and ischemia [95]. Investigations involving mammals revealed that exposure of nanoparticles to the cardiovascular system adversely affects heart function [96]. An example of such nanosized particles is MPs, internalized in cardiomyocytes, leading to injury and apoptosis in the myocardium. It increases myocardial cardiac Troponin I and creatine kinase-MB, two key markers of damage to the myocardium. All these factors lead to damage to vascular function, an increase in blood pressure, myocardial infarction, and fibrosis in the cardiovascular system [97]. Fibrosis is characterized by the deposition of fibrous connective tissue in the myocardium due to injury and chronic inflammation. This results in a decrease in cardiac output and function and an increased risk of heart failure.

### 5.2 Gastrointestinal tract

**Fig. 1:**
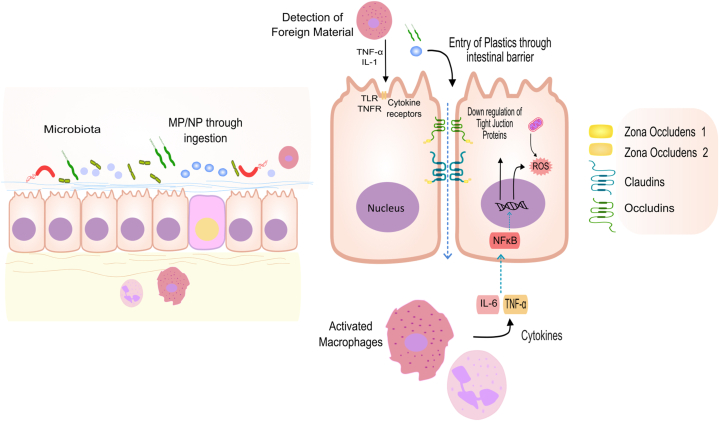
**Possible pathway of microplastics for crossing the intestinal barrier further causing pathogenic invasion.** Detection of foreign particles by immune cells and their activation leads to the release of cytokines that increase cell permeability, resulting in the passage of MPs and NPs through the intestinal barrier. By crossing the intestinal barrier, microplastics damage the tight junction among the cells characterized by the breakdown of junctional proteins such as zonula occludens (ZO-1 and ZO-2), claudins, and occludins. The breakdown of the intestinal barrier, along with microbial infections, can lead to the activation of ROS-induced cytokine production, ultimately helping the pathogen to elicit a robust immune response, thereby aggravating the disease pathogenesis.

The epithelium in the gut is the human body's largest mucosal surface, marking the first line of defense for the internal environment and the intestinal lumen. It is reported that MPs <150 μm may translocate across the gut epithelium, but the absorption may only be limited to ≤0.3 % [98]. Moreover, there is a possibility that smaller particles (about 5 μm or less) can be taken up inside the intestinal cells, and even smaller particles (<1.5 μm or more likely those in the nano-scale) could even be distributed systemically and accumulate in organs [98,99]. The interaction between the epithelium and MPs results in the deformation and disorganization of the former in terrestrial models [100]. In mice, exposure to MPs damaged the cell-to-cell integrity of the intestinal wall due to a decrease in transcription levels of tight junction proteins (Claudin-1 and ZO-1), and damage to the mucus barrier has also been reported [101,102] (Fig. 1).

The gut microbiota is an aggregation of up to 100 trillion symbiotic bacteria that live in the human gut. By producing a variety of substances, including vitamins, short-chain fatty acids, and health-beneficial products such as anti-inflammatory, analgesic, and antioxidant products, the active gut microbiota can regulate gene expression, promote digestion, and influence human metabolic and immune processes [103]. The dysbiosis of this microflora is defined as an imbalance of gut microbes leading to unhealthy consequences; the cause of this imbalance can be the uninhibited growth of bad bacteria such as *Fusobacterium nucleatum*, *Helicobacter pylori*, *Clostridium perfringens,* etc. [104]. Studies have reported that the diversity and composition of gut microbiota can be affected by plastic particles. In other animal models, exposure to MPs and NPs has resulted in the loss of populations of *Proteobacteria*, *Actinobacteria*, *Firmicutes*, and *Bacteroides* [101,105]. MPs' impact on the human gut microbiota has not yet been studied extensively; nonetheless, it may provide crucial evidence on diseases caused by microbiome dysbiosis, such as diabetes, cardiovascular disease, and colon cancer [[106], [107], [108], [109]]. Lastly, the potential of plastics to instigate an inflammatory response that can lead to the migration of pathogens or their secretory molecules through circulation and cause ailments in various other organ systems is yet to be studied.

### 5.3 Nervous system

The blood-brain barrier is a critical secondary barrier responsible for maintaining brain tissue homeostasis by restricting the entry of harmful substances. The presence of plastics in the brain and other components of the nervous system has been extensively studied in marine organisms but nominally in mammalian models [101,[110], [111], [112]]. Nonetheless, small-sized particles of plastics sized ≤100 μm can cross the blood-brain barrier, causing neurotoxicity. Jin et al. reported in mice that the MPs & NPs disrupt the BBB, attributing to the disruption of tight junctions and altered levels of inflammatory cytokines. Additionally, their presence can harm inflammation-related oxidative stress that can be attributed to the higher levels of apoptotic proteins and inhibit the acetylcholinesterase (AChE) enzyme [113]. The enzyme AChE is responsible for proper acetylcholine breakdown and, thus, liable for sound nerve signal transmission [114,115]. The inhibition of this enzyme may lead to overexcitation of the neurons. The result of this overexcitation is direct injury to the neuronal network and the introduction and/or exacerbation of neuronal disorders, including Alzheimer's disease, multiple sclerosis, etc.

## 6 Effect on microbiome, inflammation and biofilm formation

**Fig. 2:**
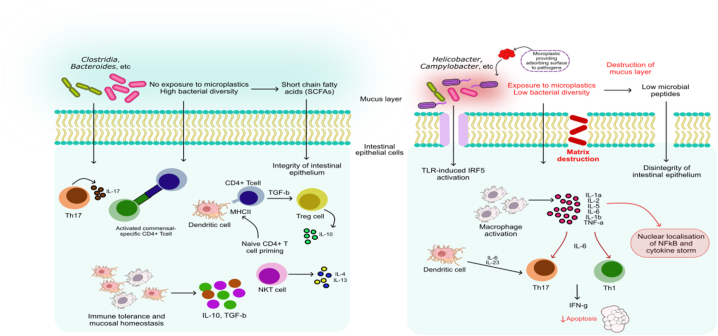
**Schematic representation of signaling cascade of microplastic and pathogen in host intestinal cells (Left panel: typical pathogenesis of microbes, Right panel: effect of microplastics on pathogenesis).** When microbial homeostasis is maintained, the host cells maintain immune tolerance against the pathogenic microbes. Meanwhile, once the host microenvironment gets exposed to microplastics, the microbiota disrupts the immune homeostasis and activates the cascade to secrete pro-inflammatory cytokines. These cytokines subsequently disrupt the membrane permeability of intestinal epithelial cells. Moreover, the microplastic surface enhances the bioavailability of microbial secretions.

In recent years, the studies on the effect of MPs and NPs infiltration in the GIT of organisms including humans have shown a decline of beneficial bacteria, such as Bacteroidetes (*Bacteroides*, *Parabacteroides*, *Dysgonomonas*, *Clostridiales*, *Akkermansia*, *Alistipes*, and *Muribaculum*), Erysipelatoclostridiaceae, Butyricicoccaceae, Actinobacteria (*Bifidobacterium* spp.) Lacetospirillum and Lactobacillus. Meanwhile, an increment has occurred in the commensal and pathogenic bacteria, such as Firmicutes (*Bacillaceae*, *Staphylococcus*, *Lactococcus*, *Phascolarctobacterium*, *Lachnoclostridium* and *Megasphaera*), Cyanobacteria, Desulfobacterota (*Bilophila*), Fusobacteria, Melainabacteria, Proteobacteria (*Acinetobacter*, *Neisseria*, *Legionella*, *Pseudomonas*, *Ottowia*, *Vibrio*, *Haemophilus*, *Methyloversatilis* and *Polynucleobacter*) [[116], [117], [118], [119], [120], [121], [122]]. The direct effect of plastics on microbial diversity can be observed as the dysbiosis and the subsequent acute inflammation and lipid metabolism disorder described by Li et al. and Lu et al. in mice. Moreover, according to the predictions of Jin et al. using the Kyoto Encyclopedia of Genes and Genomes (KEGG) metabolic pathways database, MPs influence functional genes of main microbial metabolic pathways (e.g. pyruvate and tyrosine metabolisms), fatty acid biosynthesis and genes-related to bacterial invasion of epithelial cells. Interestingly, the gut microbiome has also been reported to degrade larger microplastics into smaller particles through plastic-degrading enzymes to increase their ability to pass through the intestinal barrier [123]. The microbes in the gastrointestinal tract have also been known to resist the introduction of foreign materials by activating and regulating signal transduction pathways, such as the TLR pathway. Many pro-inflammatory transcriptional factors, such as Activator protein 1 (AP-1) and Interferon regulatory factor 4 (IRF4) activate proinflammatory cytokines such as IL-6, IL-8, TNF- α, etc. to exacerbate intestinal inflammation (Fig. 2) [124]. The study by Nugrahapraja et al. indicated the prevalence of the *feaB* gene (this gene encodes phenylacetaldehyde dehydrogenase, which is usually present in styrene-degrading bacteria) followed by other plastic-degrading genes, such as polyurethenase and esterase. Additionally, exacerbation of existing physiological disorders has also been reported by Zheng et al. Their observations involving mice with induced ulcerative colitis included amplified inflammation marked by increased levels of IL-1β, TNF-α and IFN-γ; promotion in the differentiation of adipocytes; increased peroxidation levels of liver lipids and enhanced liver metabolic disorders in mice with colitis marked by the accumulation of triglycerides and elevated levels of malondialdehyde and peroxisome proliferator-activated receptor gamma (PPAR-γ) [125]. The effects of plastic additives on the gut microbiome are not extensively documented. However, the known effects are increased bioavailability, systemic circulation and inflammation in aquatic animals and mice [126,127]. Furthermore, the pesticides that can be adsorbed by the MPs can also have a plethora of effects on gut microbes, such as exacerbation of existing conditions like gut dysbiosis, a decline in mitochondrial biomass and membrane potential, and amplification in pathogen-induced carcinogenesis (for example, *Helicobacter pylori*) [128].

**Fig. 3:**
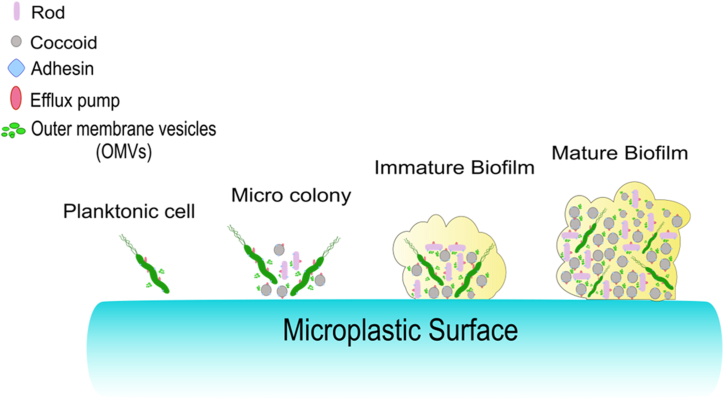
**Progression of microbial biofilm on Microplastic surface.** Various environmental triggers lead to microbes' adhesion on the microplastics' surface. The colonizing microbes further secrete outer membrane vesicles (OMVs), including signaling molecules potentially acting as a trigger to form micro-colony. Further interaction among different microbes, such as bacillus or coccoid bacteria, leads to the formation of a mature biofilm on the microplastic surfaces, acting as inducers of carcinogenesis due to the higher sustenance of pathogenic microbiota.

Microorganisms occupy numerous ecological niches in human ecosystems. The microbes in these niches range from planktonic cells to colonies, and aggregates of several colonies adhered to any inert surface called biofilms. Biofilm formation is brought about when microbes sense environmental triggers such as nutrient deprivation; for instance, in the case of gram-negative bacteria*,* low-nutrient media can trigger biofilm formation [129]. Within a biofilm, cells are protected from environmental stress, like the action of antibiotics and the host's immune system [130]. Biofilms are prominent kinds of multicellular colonies that invade human tissue surfaces. Studies have shown that plastics degrade naturally in ecosystems and inside the gut to form MPs and NPs with favorable surfaces to aid biofilm formation [[131], [132], [133], [134]]. The result of amiable conditions for biofilm formation is an increase in the bioavailability of opportunistic pathogenic microbes such as *Vibrio* spp. and *Escherichia coli* by acting as a “raft” for many substances, including microbes [[135], [136], [137]]. Studies have also found that these microplastic biofilms differ in microbial composition compared to natural substrates, such as leaf and stone. For example, two opportunistic human pathogens (*Pseudomonas monteilii* and *Pseudomonas mendocina*) have been discovered on microplastic biofilm [138]. Similarly, other opportunistic pathogens, such as *Helicobacter pylori* and *Fusobacterium* spp*,* can become highly bioavailable because of the ability of MPs and NPs to act as vehicles. *H. pylori* is a known group 1 carcinogen responsible for acute-chronic inflammation that alters cellular processes such as genomic integrity, cell cycle, autophagy, and antioxidant defense [139,140]. Moreover, such pathogens have also been linked to dysbiosis of the gut microbiome since a healthy gut microbiome helps in metabolism, neuroendocrine activity, and immunity. The consequences of this dysbiosis are various ailments, including irritable bowel syndrome, leaky gut syndrome, and neurological disorders such as Parkinson's disease, multiple sclerosis, and Huntington's disease [141]. Furthermore, in biological scenarios, the biofilm formation activity is reported as a morphological change due to the activity of protein/efflux pumps, adhesin proteins and outer membrane vesicles (OMVs), laying down the foundation of the morphological and physiological characteristics of the bacterial colony [142] (Fig. 3).

Consequently, the surface provided by the MPs & NPs for biofilm formation can increase the pathogenicity, antibiotic resistance, and carcinogenic ability of such pathogens. The effect of MPs and NPs on different parts of the gastrointestinal tract (GIT), especially the intestine, including duodenum, jejunum, ileum, and colon, has been explored; these include the dysbiosis of microbiota, oxidative stress, and inflammation [99,117]. Moreover, gram-positive bacteria such as *Bacillus subtilis* have been observed to form biofilm and promote the natural transformation of extracellular antibiotic resistance genes (ARGs) at the single-cell and multi-species levels, compared to planktonic forms [143]. Therefore, the plastic surface can also act as a hotspot for horizontal gene transfer in addition to acting as a crucial niche.

Although both the pathogenic microbes and MPs are present in the same environment, available literature rarely explores their correlation in the progression or amplification of pathologies presented by the individual effects of both pathogenic microbes and plastics.

## 7 Conclusion

The widespread use of plastics has resulted in an alarming increase in plastic waste, forming MPs and NPs. These minute plastic particles enter organisms by inhalation, ingestion, and direct contact. While the harmful effects of plastics on aquatic ecosystems and species are well documented, evidence of their consequences on human health needs to be clarified. MPs enter the human body predominantly through the ingestion of aquatic animals, with additional routes of exposure including contaminated crops, beverages, cosmetics, and pesticides. Despite the original hypothesis that the liver was the predominant location of MP accumulation, newer research has identified the gastrointestinal tract and lymph nodes, particularly the small intestine, as the key retention sites. Inflammation, intestinal epithelial damage, intestinal barrier disruption, and gut microbial dysbiosis are among the documented outcomes. Furthermore, the surface of MPs acts as a potential carrier for pollutants and poisons, contributing to gut dysbiosis and enabling opportunistic pathogen invasion. While clear evidence of plastics' direct influence on human health is still lacking, the proven presence of micro- and nano-scale plastics in human tissues and feces strongly suggests their positive ingestion and accumulation. As a result, the link between gastrointestinal dysregulation and plastics in the human gut is very conceivable, necessitating additional research and public awareness to mitigate the potential health hazards connected with plastic pollution.

## CRediT authorship contribution statement

**Pramesh Sinha:** Writing – original draft, Visualization, Validation, Software, Resources, Methodology, Investigation, Formal analysis, Data curation, Conceptualization. **Vaishali Saini:** Writing – review & editing, Visualization, Validation, Resources, Methodology, Investigation, Formal analysis, Data curation. **Nidhi Varshney:** Writing – review & editing, Visualization, Validation, Resources, Methodology, Formal analysis, Data curation. **Rajan Kumar Pandey:** Writing – review & editing, Visualization, Validation. **Hem Chandra Jha:** Writing – review & editing, Visualization, Validation, Supervision, Software, Resources, Project administration, Investigation, Funding acquisition, Formal analysis, Data curation, Conceptualization.

## Declaration of competing interest

The authors declare that they have no known competing financial interests or personal relationships that could have appeared to influence the work reported in this paper.

## References

[1] Mutha N.H., Patel M., Premnath V. (2006). Plastics materials flow analysis for India. Resour. Conserv. Recycl..

[2] Laskar N., Kumar U. (2019). Plastics and microplastics: a threat to environment. Environ. Technol. Innovat..

[3] Barnes D.K.A., Galgani F., Thompson R.C., Barlaz M. (2009). Accumulation and fragmentation of plastic debris in global environments. Phil. Trans. Biol. Sci..

[4] Cai H., Xu E.G., Du F., Li R., Liu J., Shi H. (2021). Analysis of environmental nanoplastics: progress and challenges. Chem. Eng. J..

[5] Hidalgo-Ruz V., Gutow L., Thompson R.C., Thiel M. (2012). Microplastics in the marine environment: a review of the methods used for identification and quantification. Environ. Sci. Technol..

[6] Kosuth M., Mason S.A., Wattenberg E.V. (2018). Anthropogenic contamination of tap water, beer, and sea salt. PLoS One.

[7] Sarkar D.J., Das Sarkar S., Mukherjee S., Das B.K., Kumar M., Snow D.D., Honda R., Mukherjee S. (2021). Contaminants in Drinking and Wastewater Sources: Challenges and Reigning Technologies.

[8] Goswami P., Vinithkumar N.V., Dharani G. (2020). First evidence of microplastics bioaccumulation by marine organisms in the port blair bay, andaman Islands. Mar. Pollut. Bull..

[9] Li R., Nie J., Qiu D., Li S., Sun Y., Wang C. (2023). Toxic effect of chronic exposure to polyethylene nano/microplastics on oxidative stress, neurotoxicity and gut microbiota of adult zebrafish (Danio rerio). Chemosphere.

[10] Domenech J., Marcos R. (2021). Pathways of human exposure to microplastics, and estimation of the total burden. Curr. Opin. Food Sci..

[11] Kumar R., Manna C., Padha S., Verma A., Sharma P., Dhar A., Ghosh A., Bhattacharya P. (2022). Micro(nano)plastics pollution and human health: how plastics can induce carcinogenesis to humans?. Chemosphere.

[12] Guerranti C., Martellini T., Perra G., Scopetani C., Cincinelli A. (2019). Microplastics in cosmetics: environmental issues and needs for global bans. Environ. Toxicol. Pharmacol..

[13] Deng Y., Zhang Y., Lemos B., Ren H. (2017). Tissue accumulation of microplastics in mice and biomarker responses suggest widespread health risks of exposure. Sci. Rep..

[14] Ribeiro F., O'Brien J.W., Galloway T., Thomas K.V. (2019). Accumulation and fate of nano- and micro-plastics and associated contaminants in organisms. TrAC, Trends Anal. Chem..

[15] Allen S., Allen D., Karbalaei S., Maselli V., Walker T.R. (2022). Micro(nano)plastics sources, fate, and effects: what we know after ten years of research. Journal of Hazardous Materials Advances.

[16] Sofield C.E., Anderton R.S., Gorecki A.M. (2024). Mind over microplastics: exploring microplastic-induced gut disruption and gut-brain-Axis consequences. Curr. Issues Mol. Biol..

[17] Teuten E.L., Rowland S.J., Galloway T.S., Thompson R.C. (2007). Potential for plastics to transport hydrophobic contaminants. Environ. Sci. Technol..

[18] Mohamed Nor N.H., Koelmans A.A. (2019). Transfer of PCBs from microplastics under simulated gut fluid conditions is biphasic and reversible. Environ. Sci. Technol..

[19] Dieterich W., Schink M., Zopf Y. (2018). Microbiota in the gastrointestinal tract. Med. Sci..

[20] Carding S., Verbeke K., Vipond D.T., Corfe B.M., Owen L.J. (2015). Dysbiosis of the gut microbiota in disease. Microb. Ecol. Health Dis..

[21] Al-Malaika S., Axtell F., Rothon R., Gilbert M., Gilbert M. (2017). Brydson's Plastics Materials.

[22] Murphy J. (2001).

[23] Hermabessiere L., Dehaut A., Paul-Pont I., Lacroix C., Jezequel R., Soudant P., Duflos G. (2017). Occurrence and effects of plastic additives on marine environments and organisms: a review. Chemosphere.

[24] Hasegawa T., Mizukawa K., Yeo B.G., Sekioka T., Takada H., Nakaoka M. (2022). The significance of trophic transfer of microplastics in the accumulation of plastic additives in fish: an experimental study using brominated flame retardants and UV stabilizers. Mar. Pollut. Bull..

[25] Witchey S.K., Sutherland V., Collins B., Roberts G., Shockley K.R., Vallant M., Krause J., Cunny H., Waidyanatha S., Mylchreest E., Sparrow B., Moyer R., Behl M. (2023). Reproductive and developmental toxicity following exposure to organophosphate ester flame retardants and plasticizers, triphenyl phosphate and isopropylated phenyl phosphate, in Sprague Dawley rats. Toxicol. Sci..

[26] Alijagic A., Suljević D., Fočak M., Sulejmanović J., Šehović E., Särndahl E., Engwall M. (2024). The triple exposure nexus of microplastic particles, plastic-associated chemicals, and environmental pollutants from a human health perspective. Environ. Int..

[27] Kumari A., Rajput V.D., Mandzhieva S.S., Rajput S., Minkina T., Kaur R., Sushkova S., Kumari P., Ranjan A., Kalinitchenko V.P., Glinushkin A.P. (2022). Microplastic pollution: an emerging threat to terrestrial plants and insights into its remediation strategies. Plants.

[28] Gunaalan K., Fabbri E., Capolupo M. (2020). The hidden threat of plastic leachates: a critical review on their impacts on aquatic organisms. Water Res..

[29] Fuller S., Gautam A. (2016). A procedure for measuring microplastics using pressurized fluid extraction. Environ. Sci. Technol..

[30] Zhu F., Zhu C., Wang C., Gu C. (2019). Occurrence and ecological impacts of microplastics in soil systems: a review. Bull. Environ. Contam. Toxicol..

[31] Yang H., Yumeng Y., Yu Y., Yinglin H., Fu B., Wang J. (2022). Distribution, sources, migration, influence and analytical methods of microplastics in soil ecosystems. Ecotoxicol. Environ. Saf..

[32] Wang W., Yuan W., Xu E.G., Li L., Zhang H., Yang Y. (2022). Uptake, translocation, and biological impacts of micro(nano)plastics in terrestrial plants: progress and prospects. Environ. Res..

[33] Dietz K.-J., Herth S. (2011). Plant nanotoxicology. Trends Plant Sci..

[34] Rong S., Wang S., Liu H., Li Y., Huang J., Wang W., Han B., Su S., Liu W. (2024). Evidence for the transportation of aggregated microplastics in the symplast pathway of oilseed rape roots and their impact on plant growth. Sci. Total Environ..

[35] González-Morales S., Parera C.A., Juárez-Maldonado A., la Fuente M.C.D., Benavides-Mendoza A., Husen A., Jawaid M. (2020). Nanomaterials for Agriculture and Forestry Applications.

[36] Li L., Luo Y., Li R., Zhou Q., Peijnenburg W.J.G.M., Yin N., Yang J., Tu C., Zhang Y. (2020). Effective uptake of submicrometre plastics by crop plants via a crack-entry mode. Nat. Sustain..

[37] Xu Z., Zhang Y., Lin L., Wang L., Sun W., Liu C., Yu G., Yu J., Lv Y., Chen J., Chen X., Fu L., Wang Y. (2022). Toxic effects of microplastics in plants depend more by their surface functional groups than just accumulation contents. Sci. Total Environ..

[38] Bosker T., Bouwman L.J., Brun N.R., Behrens P., Vijver M.G. (2019). Microplastics accumulate on pores in seed capsule and delay germination and root growth of the terrestrial vascular plant Lepidium sativum. Chemosphere.

[39] Schirmel J., Albert J., Kurtz M.P., Muñoz K. (2018). Plasticulture changes soil invertebrate assemblages of strawberry fields and decreases diversity and soil microbial activity. Appl. Soil Ecol..

[40] Guo M., Zhao F., Tian L., Ni K., Lu Y., Borah P. (2022). Effects of polystyrene microplastics on the seed germination of herbaceous ornamental plants. Sci. Total Environ..

[41] Colzi I., Renna L., Bianchi E., Castellani M.B., Coppi A., Pignattelli S., Loppi S., Gonnelli C. (2022). Impact of microplastics on growth, photosynthesis and essential elements in Cucurbita pepo L. J. Hazard Mater..

[42] Liu Y., Guo R., Zhang S., Sun Y., Wang F. (2022). Uptake and translocation of nano/microplastics by rice seedlings: evidence from a hydroponic experiment. J. Hazard Mater..

[43] Khalid N., Aqeel M., Noman A. (2020). Microplastics could be a threat to plants in terrestrial systems directly or indirectly. Environ. Pollut..

[44] de Souza Machado A.A., Lau C.W., Kloas W., Bergmann J., Bachelier J.B., Faltin E., Becker R., Görlich A.S., Rillig M.C. (2019). Microplastics can change soil properties and affect plant performance. Environ. Sci. Technol..

[45] Stock V., Böhmert L., Lisicki E., Block R., Cara-Carmona J., Pack L.K., Selb R., Lichtenstein D., Voss L., Henderson C.J., Zabinsky E., Sieg H., Braeuning A., Lampen A. (2019). Uptake and effects of orally ingested polystyrene microplastic particles in vitro and in vivo. Arch. Toxicol..

[46] Moore C.J. (2008). Synthetic polymers in the marine environment: a rapidly increasing, long-term threat. Environ. Res..

[47] Lusher A., Bergmann M., Gutow L., Klages M. (2015). Marine Anthropogenic Litter.

[48] Lusher A.L., O'Donnell C., Officer R., O'Connor I. (2016). Microplastic interactions with North Atlantic mesopelagic fish. ICES (Int. Counc. Explor. Sea) J. Mar. Sci..

[49] Alomar C., Deudero S. (2017). Evidence of microplastic ingestion in the shark Galeus melastomus Rafinesque, 1810 in the continental shelf off the western Mediterranean Sea. Environ. Pollut..

[50] Wright S.L., Kelly F.J. (2017). Plastic and human health: a micro issue?. Environ. Sci. Technol..

[51] Abbasi S., Soltani N., Keshavarzi B., Moore F., Turner A., Hassanaghaei M. (2018). Microplastics in different tissues of fish and prawn from the Musa Estuary, Persian Gulf. Chemosphere.

[52] Vagner M., Boudry G., Courcot L., Vincent D., Dehaut A., Duflos G., Huvet A., Tallec K., Zambonino-Infante J.-L. (2022). Experimental evidence that polystyrene nanoplastics cross the intestinal barrier of European seabass. Environ. Int..

[53] Wright S.L., Thompson R.C., Galloway T.S. (2013). The physical impacts of microplastics on marine organisms: a review. Environ. Pollut..

[54] Cole M., Lindeque P., Halsband C., Galloway T.S. (2011). Microplastics as contaminants in the marine environment: a review. Mar. Pollut. Bull..

[55] Greven A.-C., Merk T., Karagöz F., Mohr K., Klapper M., Jovanović B., Palić D. (2016). Polycarbonate and polystyrene nanoplastic particles act as stressors to the innate immune system of fathead minnow (Pimephales promelas). Environ. Toxicol. Chem..

[56] Lu Y., Zhang Y., Deng Y., Jiang W., Zhao Y., Geng J., Ding L., Ren H. (2016). Uptake and accumulation of polystyrene microplastics in zebrafish (Danio rerio) and toxic effects in liver. Environ. Sci. Technol..

[57] Carlos de Sá L., Luís L.G., Guilhermino L. (2015). Effects of microplastics on juveniles of the common goby (Pomatoschistus microps): confusion with prey, reduction of the predatory performance and efficiency, and possible influence of developmental conditions. Environ. Pollut..

[58] Kentin E. (2018). Banning microplastics in cosmetic products in europe: legal challenges. Springer Water.

[59] Cox K.D., Covernton G.A., Davies H.L., Dower J.F., Juanes F., Dudas S.E. (2019). Human consumption of microplastics. Environ. Sci. Technol..

[60] Li P., Liu J. (2024). Micro(nano)plastics in the human body: sources, occurrences, fates, and health risks. Environ. Sci. Technol..

[61] Vethaak A.D., Leslie H.A. (2016). Plastic debris is a human health issue. Environ. Sci. Technol..

[62] Leslie H.A., van Velzen M.J.M., Brandsma S.H., Vethaak A.D., Garcia-Vallejo J.J., Lamoree M.H. (2022). Discovery and quantification of plastic particle pollution in human blood. Environ. Int..

[63] Chambers E., Mitragotri S. (2004). Prolonged circulation of large polymeric nanoparticles by non-covalent adsorption on erythrocytes. J. Contr. Release.

[64] Anselmo A.C., Gupta V., Zern B.J., Pan D., Zakrewsky M., Muzykantov V., Mitragotri S. (2013). Delivering nanoparticles to lungs while avoiding liver and spleen through adsorption on red blood cells. ACS Nano.

[65] Yang C.-S., Chang C.-H., Tsai P.-J., Chen W.-Y., Tseng F.-G., Lo L.-W. (2004). Nanoparticle-based in vivo investigation on Blood−Brain barrier permeability following ischemia and reperfusion. Anal. Chem..

[66] Grafmueller S., Manser P., Diener L., Diener P.-A., Maeder-Althaus X., Maurizi L., Jochum W., Krug H.F., Buerki-Thurnherr T., von Mandach U., Wick P. (2015). Bidirectional transfer study of polystyrene nanoparticles across the placental barrier in an ex vivo human placental perfusion model. Environ. Health Perspect..

[67] Schwabl P., Köppel S., Königshofer P., Bucsics T., Trauner M., Reiberger T., Liebmann B. (2019). Detection of various microplastics in human stool. Ann. Intern. Med..

[68] Zhang J., Wang L., Trasande L., Kannan K. (2021). Occurrence of polyethylene terephthalate and polycarbonate microplastics in infant and adult feces. Environ. Sci. Technol. Lett..

[69] Pauly J.L., Stegmeier S.J., Allaart H.A., Cheney R.T., Zhang P.J., Mayer A.G., Streck R.J. (1998). Inhaled cellulosic and plastic fibers found in human lung tissue. Cancer Epidemiol. Biomarkers Prev..

[70] Smith M., Love D.C., Rochman C.M., Neff R.A. (2018). Microplastics in seafood and the implications for human health. Curr Envir Health Rpt.

[71] Vikas Madhav N., Gopinath K.P., Krishnan A., Rajendran N., Krishnan A. (2020). A critical review on various trophic transfer routes of microplastics in the context of the Indian coastal ecosystem. Watershed Ecology and the Environment.

[72] Fournier E., Leveque M., Ruiz P., Ratel J., Durif C., Chalancon S., Amiard F., Edely M., Bezirard V., Gaultier E., Lamas B., Houdeau E., Lagarde F., Engel E., Etienne-Mesmin L., Blanquet-Diot S., Mercier-Bonin M. (2023). Microplastics: what happens in the human digestive tract? First evidences in adults using in vitro gut models. J. Hazard Mater..

[73] Tong X., Li B., Li J., Li L., Zhang R., Du Y., Zhang Y. (2022). Polyethylene microplastics cooperate with Helicobacter pylori to promote gastric injury and inflammation in mice. Chemosphere.

[74] Chelombitko M.A. (2018). Role of reactive oxygen species in inflammation: a minireview. Mosc. Univ. Biol. Sci. Bull..

[75] Chen J., Chen X., Xuan Y., Shen H., Tang Y., Zhang T., Xu J. (2023). Surface functionalization-dependent inflammatory potential of polystyrene nanoplastics through the activation of MAPK/NF-κB signaling pathways in macrophage Raw 264.7. Ecotoxicol. Environ. Saf..

[76] Tu C., Chen T., Zhou Q., Liu Y., Wei J., Waniek J.J., Luo Y. (2020). Biofilm formation and its influences on the properties of microplastics as affected by exposure time and depth in the seawater. Sci. Total Environ..

[77] Bhagwat G., Tran T.K.A., Lamb D., Senathirajah K., Grainge I., O'Connor W., Juhasz A., Palanisami T. (2021). Biofilms enhance the adsorption of toxic contaminants on plastic microfibers under environmentally relevant conditions. Environ. Sci. Technol..

[78] Tziourrou P., Bourikas K., Karapanagioti H.K., Cocca M., Di Pace E., Errico M.E., Gentile G., Montarsolo A., Mossotti R., Avella M. (2020). Proceedings of the 2nd International Conference on Microplastic Pollution in the Mediterranean Sea.

[79] da Costa J.P., Santos P.S.M., Duarte A.C., Rocha-Santos T. (2016). (Nano)plastics in the environment – sources, fates and effects. Sci. Total Environ..

[80] Shaw I.C., Jones H.B. (1994). Shaw and Jones reply: the multifactorial nature of carcinogenesis. Trends Pharmacol. Sci..

[81] Ahmed M.B., Rahman MdS., Alom J., Hasan M.D.S., Johir M.A.H., Mondal M.I.H., Lee D.-Y., Park J., Zhou J.L., Yoon M.-H. (2021). Microplastic particles in the aquatic environment: a systematic review. Sci. Total Environ..

[82] Yee M.S.-L., Hii L.-W., Looi C.K., Lim W.-M., Wong S.-F., Kok Y.-Y., Tan B.-K., Wong C.-Y., Leong C.-O. (2021). Impact of microplastics and nanoplastics on human health. Nanomaterials.

[83] Yin K., Wang Y., Zhao H., Wang D., Guo M., Mu M., Liu Y., Nie X., Li B., Li J., Xing M. (2021). A comparative review of microplastics and nanoplastics: toxicity hazards on digestive, reproductive and nervous system. Sci. Total Environ..

[84] Elsaesser A., Howard C.V. (2012). Toxicology of nanoparticles. Adv. Drug Deliv. Rev..

[85] Forte M., Iachetta G., Tussellino M., Carotenuto R., Prisco M., De Falco M., Laforgia V., Valiante S. (2016). Polystyrene nanoparticles internalization in human gastric adenocarcinoma cells. Toxicol. Vitro.

[86] Walczak A.P., Kramer E., Hendriksen P.J.M., Tromp P., Helsper J.P.F.G., van der Zande M., Rietjens I.M.C.M., Bouwmeester H. (2015). Translocation of differently sized and charged polystyrene nanoparticles in in vitro intestinal cell models of increasing complexity. Nanotoxicology.

[87] Baj J., Dring J.C., Czeczelewski M., Kozyra P., Forma A., Flieger J., Kowalska B., Buszewicz G., Teresiński G. (2022). Derivatives of plastics as potential carcinogenic factors: the current state of knowledge. Cancers.

[88] McCarthy J., Gong X., Nahirney D., Duszyk M., Radomski M. (2011). Polystyrene nanoparticles activate ion transport in human airway epithelial cells. Int. J. Nanomed..

[89] Fuchs A.-K., Syrovets T., Haas K.A., Loos C., Musyanovych A., Mailänder V., Landfester K., Simmet T. (2016). Carboxyl- and amino-functionalized polystyrene nanoparticles differentially affect the polarization profile of M1 and M2 macrophage subsets. Biomaterials.

[90] Ding R., Chen Y., Shi X., Li Y., Yu Y., Sun Z., Duan J. (2024). Size-dependent toxicity of polystyrene microplastics on the gastrointestinal tract: oxidative stress related-DNA damage and potential carcinogenicity. Sci. Total Environ..

[91] Bouwmeester H., Hollman P.C.H., Peters R.J.B. (2015). Potential health impact of environmentally released micro- and nanoplastics in the human food production chain: experiences from nanotoxicology. Environ. Sci. Technol..

[92] Deville S., Penjweini R., Smisdom N., Notelaers K., Nelissen I., Hooyberghs J., Ameloot M. (2015). Intracellular dynamics and fate of polystyrene nanoparticles in A549 Lung epithelial cells monitored by image (cross-) correlation spectroscopy and single particle tracking. Biochim. Biophys. Acta Mol. Cell Res..

[93] Wu B., Wu X., Liu S., Wang Z., Chen L. (2019). Size-dependent effects of polystyrene microplastics on cytotoxicity and efflux pump inhibition in human Caco-2 cells. Chemosphere.

[94] Goodman K.E., Hua T., Sang Q.-X.A. (2022). Effects of polystyrene microplastics on human kidney and liver cell morphology, cellular proliferation, and metabolism. ACS Omega.

[95] Yin P., Guo J., Wang L., Fan W., Lu F., Guo M., Moreno S.B.R., Wang Y., Wang H., Zhou M., Dong Z. (2020). Higher risk of cardiovascular disease associated with smaller size-fractioned particulate matter. Environ. Sci. Technol. Lett..

[96] Savi M., Rossi S., Bocchi L., Gennaccaro L., Cacciani F., Perotti A., Amidani D., Alinovi R., Goldoni M., Aliatis I., Lottici P.P., Bersani D., Campanini M., Pinelli S., Petyx M., Frati C., Gervasi A., Urbanek K., Quaini F., Buschini A., Stilli D., Rivetti C., Macchi E., Mutti A., Miragoli M., Zaniboni M. (2014). Titanium dioxide nanoparticles promote arrhythmias via a direct interaction with rat cardiac tissue. Part. Fibre Toxicol..

[97] Li Z., Zhu S., Liu Q., Wei J., Jin Y., Wang X., Zhang L. (2020). Polystyrene microplastics cause cardiac fibrosis by activating Wnt/β-catenin signaling pathway and promoting cardiomyocyte apoptosis in rats. Environ. Pollut..

[98] E.P. on C. in the F. Chain (2016). CONTAM), Presence of microplastics and nanoplastics in food, with particular focus on seafood. EFSA J..

[99] Paul M.B., Stock V., Cara-Carmona J., Lisicki E., Shopova S., Fessard V., Braeuning A., Sieg H., Böhmert L. (2020). Micro- and nanoplastics – current state of knowledge with the focus on oral uptake and toxicity. Nanoscale Adv..

[100] Jiang X., Chang Y., Zhang T., Qiao Y., Klobučar G., Li M. (2020). Toxicological effects of polystyrene microplastics on earthworm (Eisenia fetida). Environ. Pollut..

[101] Jin Y., Lu L., Tu W., Luo T., Fu Z. (2019). Impacts of polystyrene microplastic on the gut barrier, microbiota and metabolism of mice. Sci. Total Environ..

[102] Luo T., Wang C., Pan Z., Jin C., Fu Z., Jin Y. (2019). Maternal polystyrene microplastic exposure during gestation and lactation altered metabolic homeostasis in the dams and their F1 and F2 offspring. Environ. Sci. Technol..

[103] Liu B.-N., Liu X.-T., Liang Z.-H., Wang J.-H. (2021). Gut microbiota in obesity. World J. Gastroenterol..

[104] Martinez J.E., Kahana D.D., Ghuman S., Wilson H.P., Wilson J., Kim S.C.J., Lagishetty V., Jacobs J.P., Sinha-Hikim A.P., Friedman T.C. (2021). Unhealthy lifestyle and gut dysbiosis: a better understanding of the effects of poor diet and nicotine on the intestinal microbiome. Front. Endocrinol..

[105] Zhang X., Wen K., Ding D., Liu J., Lei Z., Chen X., Ye G., Zhang J., Shen H., Yan C., Dong S., Huang Q., Lin Y. (2021). Size-dependent adverse effects of microplastics on intestinal microbiota and metabolic homeostasis in the marine medaka (Oryzias melastigma). Environ. Int..

[106] Blandino G., Inturri R., Lazzara F., Di Rosa M., Malaguarnera L. (2016). Impact of gut microbiota on diabetes mellitus. Diabetes Metabol..

[107] Kitai T., Tang W.H.W. (2017). Impacto de la microbiota intestinal en la enfermedad cardiovascular. Rev. Española Cardiol..

[108] Oke S., Martin A. (2017). Insights into the role of the intestinal microbiota in colon cancer. Therap Adv Gastroenterol.

[109] Velmurugan G., Ramprasath T., Gilles M., Swaminathan K., Ramasamy S. (2017). Gut microbiota, endocrine-disrupting chemicals, and the diabetes epidemic. Trends Endocrinol. Metabol..

[110] Chen H., Hua X., Yang Y., Wang C., Jin L., Dong C., Chang Z., Ding P., Xiang M., Li H., Yu Y. (2021). Chronic exposure to UV-aged microplastics induces neurotoxicity by affecting dopamine, glutamate, and serotonin neurotransmission in Caenorhabditis elegans. J. Hazard Mater..

[111] Lei L., Liu M., Song Y., Lu S., Hu J., Cao C., Xie B., Shi H., He D. (2018). Polystyrene (nano)microplastics cause size-dependent neurotoxicity, oxidative damage and other adverse effects in Caenorhabditis elegans. Environ. Sci. Nano.

[112] Ribeiro F., Garcia A.R., Pereira B.P., Fonseca M., Mestre N.C., Fonseca T.G., Ilharco L.M., Bebianno M.J. (2017). Microplastics effects in Scrobicularia plana. Mar. Pollut. Bull..

[113] Jin H., Yang C., Jiang C., Li L., Pan M., Li D., Han X., Ding J. (2022). Evaluation of neurotoxicity in BALB/c mice following chronic exposure to polystyrene microplastics. Environ. Health Perspect..

[114] Eom H.-J., Nam S.-E., Rhee J.-S. (2020). Polystyrene microplastics induce mortality through acute cell stress and inhibition of cholinergic activity in a brine shrimp. Mol. Cell. Toxicol..

[115] Richter N., Beckers N., Onur O.A., Dietlein M., Tittgemeyer M., Kracht L., Neumaier B., Fink G.R., Kukolja J. (2018). Effect of cholinergic treatment depends on cholinergic integrity in early Alzheimer's disease. Brain.

[116] Tamargo A., Molinero N., Reinosa J.J., Alcolea-Rodriguez V., Portela R., Bañares M.A., Fernández J.F., Moreno-Arribas M.V. (2022). PET microplastics affect human gut microbiota communities during simulated gastrointestinal digestion, first evidence of plausible polymer biodegradation during human digestion. Sci. Rep..

[117] Lu L., Wan Z., Luo T., Fu Z., Jin Y. (2018). Polystyrene microplastics induce gut microbiota dysbiosis and hepatic lipid metabolism disorder in mice. Sci. Total Environ..

[118] Li B., Ding Y., Cheng X., Sheng D., Xu Z., Rong Q., Wu Y., Zhao H., Ji X., Zhang Y. (2020). Polyethylene microplastics affect the distribution of gut microbiota and inflammation development in mice. Chemosphere.

[119] Wan Z., Wang C., Zhou J., Shen M., Wang X., Fu Z., Jin Y. (2019). Effects of polystyrene microplastics on the composition of the microbiome and metabolism in larval zebrafish. Chemosphere.

[120] Yin K., Wang D., Zhang Y., Lu H., Wang Y., Xing M. (2023). Dose-effect of polystyrene microplastics on digestive toxicity in chickens (Gallus gallus): multi-omics reveals critical role of gut-liver axis. J. Adv. Res..

[121] Zhu D., Chen Q.-L., An X.-L., Yang X.-R., Christie P., Ke X., Wu L.-H., Zhu Y.-G. (2018). Exposure of soil collembolans to microplastics perturbs their gut microbiota and alters their isotopic composition. Soil Biol. Biochem..

[122] Eichinger J., Tretola M., Seifert J., Brugger D. (2024). Review: interactions between microplastics and the gastrointestinal microbiome. Ital. J. Anim. Sci..

[123] Nugrahapraja H., Sugiyo P.W.W., Putri B.Q., Ni’matuzahroh Fatimah, Huang L., Hafza N., Götz F., Santoso H., Wibowo A.T., Luqman A. (2022). Effects of microplastic on human gut microbiome: detection of plastic-degrading genes in human gut exposed to microplastics—preliminary study. Environments.

[124] Ghosal S., Bag S., Rao S.R., Bhowmik S. (2024). Exposure to polyethylene microplastics exacerbate inflammatory bowel disease tightly associated with intestinal gut microflora. RSC Adv..

[125] Zheng H., Wang J., Wei X., Chang L., Liu S. (2021). Proinflammatory properties and lipid disturbance of polystyrene microplastics in the livers of mice with acute colitis. Sci. Total Environ..

[126] Claus S.P., Guillou H., Ellero-Simatos S. (2016). The gut microbiota: a major player in the toxicity of environmental pollutants?. Npj Biofilms Microbiomes.

[127] Zhang J., Walker M.E., Sanidad K.Z., Zhang H., Liang Y., Zhao E., Chacon-Vargas K., Yeliseyev V., Parsonnet J., Haggerty T.D., Wang G., Simpson J.B., Jariwala P.B., Beaty V.V., Yang J., Yang H., Panigrahy A., Minter L.M., Kim D., Gibbons J.G., Liu L., Li Z., Xiao H., Borlandelli V., Overkleeft H.S., Cloer E.W., Major M.B., Goldfarb D., Cai Z., Redinbo M.R., Zhang G. (2022). Microbial enzymes induce colitis by reactivating triclosan in the mouse gastrointestinal tract. Nat. Commun..

[128] Singh S., Varshney N., Singothu S., Bhandari V., Jha H.C. (2024). Influence of chlorpyrifos and endosulfan and their metabolites on the virulence of *Helicobacter pylori*. Environ. Pollut..

[129] O'Toole G., Kaplan H.B., Kolter R. (2000). Biofilm Formation as microbial development. Annu. Rev. Microbiol..

[130] Hathroubi S., Zerebinski J., Ottemann K.M. (2019). Helicobacter pylori biofilm cells are metabolically distinct, express flagella, and antibiotic tolerant.

[131] Cai L., Wang J., Peng J., Wu Z., Tan X. (2018). Observation of the degradation of three types of plastic pellets exposed to UV irradiation in three different environments. Sci. Total Environ..

[132] Khoironi A., Hadiyanto H., Anggoro S., Sudarno S. (2020). Evaluation of polypropylene plastic degradation and microplastic identification in sediments at Tambak Lorok coastal area, Semarang, Indonesia. Mar. Pollut. Bull..

[133] Stock V., Fahrenson C., Thuenemann A., Dönmez M.H., Voss L., Böhmert L., Braeuning A., Lampen A., Sieg H. (2020). Impact of artificial digestion on the sizes and shapes of microplastic particles. Food Chem. Toxicol..

[134] Zhang K., Hamidian A.H., Tubić A., Zhang Y., Fang J.K.H., Wu C., Lam P.K.S. (2021). Understanding plastic degradation and microplastic formation in the environment: a review. Environ. Pollut..

[135] Kirstein I.V., Kirmizi S., Wichels A., Garin-Fernandez A., Erler R., Löder M., Gerdts G. (2016). Dangerous hitchhikers? Evidence for potentially pathogenic Vibrio spp. on microplastic particles. Mar. Environ. Res..

[136] Schmitt-Jansen M., Lips S., Schäfer H., Rummel C., Rocha-Santos T., Costa M.F., Mouneyrac C. (2022). Handbook of Microplastics in the Environment.

[137] Bowley J., Baker-Austin C., Porter A., Hartnell R., Lewis C. (2021). Oceanic hitchhikers – assessing pathogen risks from marine microplastic. Trends Microbiol..

[138] Wu X., Pan J., Li M., Li Y., Bartlam M., Wang Y. (2019). Selective enrichment of bacterial pathogens by microplastic biofilm. Water Res..

[139] Baral B., Kashyap D., Varshney N., Verma T.P., Jain A.K., Chatterji D., Kumar V., Mishra A., Kumar A., Jha H.C. (2023). Data on differential pathogenic ability of Helicobacter pylori isolated from distinct gastric niches. Data Brief.

[140] Kashyap D., Varshney N., Baral B., Kandpal M., Indari O., Jain A.K., Chatterji D., Kumar S., Parmar H.S., Sonawane A., Chandra Jha H. (2023). Helicobacter pylori infected gastric epithelial cells bypass cell death pathway through the oncoprotein Gankyrin. Advances in Cancer Biology - Metastasis.

[141] Kandpal M., Indari O., Baral B., Jakhmola S., Tiwari D., Bhandari V., Pandey R.K., Bala K., Sonawane A., Jha H.C. (2022). Dysbiosis of gut microbiota from the perspective of the gut–brain Axis: role in the provocation of neurological disorders. Metabolites.

[142] Krzyżek P., Grande R., Migdał P., Paluch E., Gościniak G. (2020). Biofilm Formation as a complex result of virulence and adaptive responses of Helicobacter pylori. Pathogens.

[143] Wang H., Xu K., Wang J., Feng C., Chen Y., Shi J., Ding Y., Deng C., Liu X. (2023). Microplastic biofilm: an important microniche that may accelerate the spread of antibiotic resistance genes via natural transformation. J. Hazard Mater..

